# SYK as a New Therapeutic Target in B-Cell Precursor Acute Lymphoblastic Leukemia

**DOI:** 10.4236/jct.2014.51015

**Published:** 2014-01

**Authors:** Fatih M. Uckun, Sanjive Qazi

**Affiliations:** 1 Department of Pediatrics, Keck School of Medicine, University of Southern California, Los Angeles, USA; 2 Children’s Center for Cancer and Blood Diseases, CHLA, Los Angeles, USA

**Keywords:** STAT3, SYK, Kinase, Phosphorylation, Apoptosis, Leukemia, Nanomedicine

## Abstract

The identification of SYK as a master regulator of apoptosis controlling the activation of the PI3-K/AKT, NF*κ*B, and STAT3 pathways—three major anti-apoptotic signaling pathways in B-lineage leukemia/lymphoma cells—prompts the hypothesis that rationally designed inhibitors targeting SYK may overcome the resistance of malignant B-lineage lymphoid cells to apoptosis and thereby provide the foundation for more effective multi-modality treatment regimens for poor prognosis B-precursor acute lymphoblastic leukemia (BPL). In recent preclinical proof-of-concept studies, a liposomal nanoparticle (LNP) formulation of a SYK substrate-binding site inhibitor, known as C61, has been developed as a nanomedicine candidate against poor prognosis and relapsed BPL. This nanoscale formulation of C61 exhibited a uniquely favorable pharmacokinetics and safety profile in mice, induced apoptosis in radiation-resistant primary leukemic cells taken directly from BPL patients as well as *in vivo* clonogenic BPL xenograft cells, destroyed the leukemic stem cell fraction of BPL blasts, and exhibited potent *in vivo* anti-leukemic activity in xenograft models of aggressive BPL. Further development of C61-LNP may provide the foundation for new and effective treatment strategies against therapy-refractory BPL.

## 1. Therapy Resistance in B-Cell Precursor Acute Lymphoblastic Leukemia

B-cell precursor acute lymphoblastic leukemia (BPL) is the most common form of cancer in children and adolescents. Despite major improvements in survival outcome of newly diagnosed BPL patients being treated on contemporary chemotherapy protocols, achieving long-term leukemia-free survival in the majority of patients who fail their frontline chemotherapy regimen and relapse remains an unmet medical need in BPL therapy [1–5]. Resistance of their leukemic clones to the pro-apoptotic effects of chemotherapy- or radiation-induced DNA double-strand breaks (DSB) hampers the attempts to improve the survival outcome of patients with relapsed BPL [6,7]. While a significant portion of patients (>80%) can be reinduced into a second remission with salvage chemotherapy protocols, the duration of such remissions is usually very short due to chemotherapy resistance of the relapse clones, especially in those patients who experience an early first relapse in their bone marrow and those who show a delayed response to their reinduction chemotherapy [1–5]. Relapse and leukemia-related mortality rates remain high even after use of very intensive radiochemotherapy regimens combined with hematopoietic stem cell transplantation (SCT) [1–5]. The results of salvage regimens for relapsed ALL are not likely to improve until we learn more about the ability of ALL blasts to survive the lethal effects of chemotherapy. A better understanding of the molecular mechanisms underlying the resistance of B-lineage leukemia cells to DSB-induced apoptosis has emerged as an exceptional focal point for translational research in modern therapy of relapsed B-lineage ALL as it may provide the foundation for more effective chemotherapy regimens. There is a paucity of data on genomic determinants of *de novo* cross-resistance to ALL chemotherapy. A better understanding of the molecular mechanisms underlying the resistance of B-lineage leukemia cells to chemotherapy-induced apoptosis may provide the foundation for more effective frontline as well as salvage regimens [8]. Furthermore, identification of reliable biomarkers of chemotherapy resistance may help identify those patient subpopulations most likely to benefit from various chemotherapy regimens. Little is known about genes that confer *de novo* cross-resistance of primary ALL cells to multiple anticancer agents, a phenotype likely associated with a poor prognosis. Multiple drug resistance can be caused by increased drug efflux mediated by transmembrane transporters such as ABCC1/MRP1, MVP/LRP, and ABCB1/MDR1 but these extensively studied multiple drug resistance mechanisms have limited clinical significance in childhood ALL. Other mechanisms of drug resistance must be operative when ALL cells exhibit *de novo* cross-resistance to multiple standard anti-leukemic agents. Several research teams have therefore embarked upon molecular target discovery efforts to identify new “druggable” targets in leukemic B-cell precursors from relapsed BPL patients using integrated multi-platform laboratory and in silico research tools [8].

Ionizing radiation as well as several chemotherapeutic agents used in BPL therapy causes DSB in nuclear DNA leading to apoptotic cell death. Both NF*κ*B and PI3-K survival pathways are activated by chemotherapeutic agents and contribute to drug resistance of cancer cells [6,7]. In the PI3-K-linked anti-apoptotic survival pathway, the serine/threonine protein kinase AKT, is also known as protein kinase B (PKB), functions downstream of PI3-K. Approximately one third of pediatric BPL cases exhibit a DNA double strand break (DSB)-resistant phenotype in vitro and chemotherapy/radiation-induced DNA DSB do not cause apoptosis in their primary leukemic cells [6,7]. Both NF*κ*B and PI3-K survival pathways are regulated by spleen tyrosine kinase (SYK [9, 10].

## 2. Spleen Tyrosine Kinase as a Master Regulator of Apoptosis

SYK regulates several signaling events in lymphoid cells [9]. Upon activation, SYK phosphorylates several signaling molecules such as SLP-65/BLNK, a “docking site” for other downstream signaling proteins and triggers a cascade of signal transduction events, which affect activation, proliferation and survival [9]. SYK is an integral part of effective pre-BCR signaling in B-cell precursors as well as BCR signaling in mature B-lymphocytes and it plays an important regulatory role in early specification and maturation events during B-cell ontogeny [9]. A rapidly growing list of downstream effectors in SYK-linked signal transduction pathways includes: PLC-*γ*, ERK2, p90RSK, RAS, GAP, MAPK, SHC, PI3 kinase (PI3-K), SHIP, CBL, and VAV [9]. SYK is an upstream regulator of the anti-apoptotic PI3-K in the BCR signaling pathway [9]. SYK can activate PI3-K via adapter proteins such as CBL, a B cell adaptor for PI3-K (BCAP), CD19 and Grb2-associated molecules, which upon SYK-mediated tyrosine phosphorylation bind to the p85 subunit of PI3-K and thereby activate it. Further, SYK regulates the anti-apoptotic mammalian target of rapamycin (mTOR) signaling pathway [9]. Notably, the anti-apoptotic NF*κ*B and STAT3 pathways are also regulated by SYK [9,11].

SYK inhibition causes disruption of important signaling pathways such as BCR signaling pathway and mTOR pathway leading to apoptotic death in leukemia and lymphoma cells [9]. On the other hand, upregulated SYK activity has been shown to cause BPL in mice [12]. An inhibitor of SYK has been reported to prevent the maturational arrest of B-cell precursors transformed by deregulated SYK activity [12]. SYK also has a BCR-independent anti-apoptotic function that is operative in human leukemic B-cell precursors corresponding to the earliest stages of human B-cell ontogeny. Most importantly, SYK inhibition causes apoptosis in primary leukemia cells from BPL patients that are resistant to chemotherapy [13].

## 3. SYK-STAT3 Molecular Complex as a Therapeutic Target in B-Precursor Acute Lymphoblastic Leukemia and Other B-Lineage Lymphoid Malignancies

The signal transducer and activator of transcription (STAT) 3 protein, a latent transcription factor which is a major component of the signaling pathways that become activated by oxidative stress (OS)-induced DNA DSB in human lymphocytes, has recently been identified as a master regulator of cell survival after exposure to DSB-induced pro-apoptotic signals [11]. Upon exposure of B-lineage lymphoid cells to radiation-induced OS, STAT3 is phosphorylated on tyrosine residue(s), forms dimers and is translocated to the nucleus [14]. In the nucleus, STAT3 acts as a transcription factor with pleiotropic downstream effects. STAT3 was reported to reduce apoptosis by suppressing OS-sensitive caspase-3 activity [15]. STAT3 activation has been shown to protect against Fas-mediated apoptotic liver injury by inhibiting caspase activities in redox-dependent and -independent mechanisms [15]. Terui *et al*. reported that STAT3 confers protection against OS in part by inactivating caspase-3 [16]. The crucial role of STAT3 in resistance to OS-induced apoptosis of B-lineage lymphoid cells was confirmed genetically using STAT3^−/−^ B cells from mice lacking STAT3 in the CD19^+^ B-cell compartment [14].

SYK has been discovered as a regulator of STAT3 and it is critical for OS-induced STAT3 activation [11]. A meta-analysis using the Oncomine database revealed a marked enrichment of the most discriminating SYK-dependent anti-apoptotic genes and confirmed STAT3 targets [11] in 18 diagnostic classes of human leukemias and lymphomas [9], of which 5 were represented in multiple studies. The message for SYK was markedly upregulated in 14 comparisons with 10 showing >2-fold differences. Our findings demonstrated that SYK can phosporylate STAT3 to induce expression of STAT3 target genes that can potentially promote the survival of malignant lymphoid cells and prevent their terminal differentiation [11]. In patients that showed upregulation of SYK, the majority (9/14) of comparisons also showed significant upregulation of at least one of the STAT3 target genes. Taken together, this meta-analysis strongly suggested that SYK activation could lead to the upregulation of anti-apoptotic genes and inhibitors of cellular differentiation in a variety of leukemias and lymphomas.

**Figure 1:**
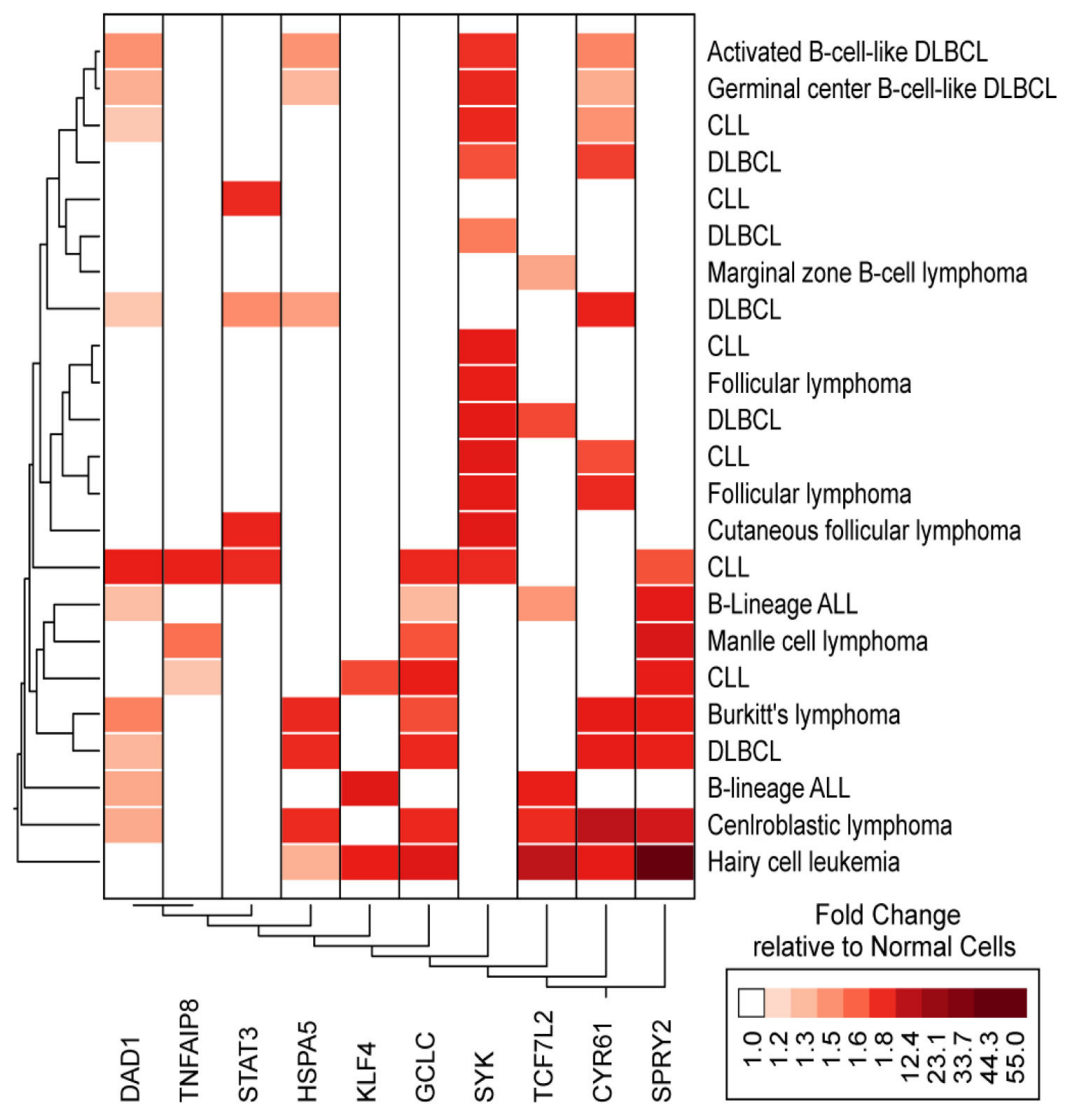
Meta-Analysis of SYK Signature Gene Expression in B-lineage Leukemia/Lymphoma Patients Using the Oncomine Database. The most consistently discriminating anti-apoptotic genes, SYK, STAT3, and confirmed STAT3 target genes [11] were cross-referenced to the Oncomine™ Research Data Base (http://www.oncomine.org/) for leukemia and lymphoma studies. Each comparison was identified by a Database reference (From the top of the cluster figure: Alizadeh Lymphoma, Alizadeh Lymphoma, Rosenwald Lymphoma, Rosenwald Multi-cancer, Valk Leukemia, Alizadeh Lymphoma, Storz Lymphoma, Rosenwald Lymphoma, Alizadeh Lymphoma, Alizadeh Lymphoma, Storz Lymphoma, Rosenwald Multi-cancer, Rosenwald Multi-cancer, Storz Lymphoma, Haslinger Leukemia, Andersson Leukemia, Basso Lymphoma, Basso Lymphoma, Basso Lymphoma, Basso Lymphoma, Maia Leukemia, Basso Lymphoma, Basso Lymphoma) and details of the normal cells used in the fold difference calculations can be found on the website. We filtered fold difference relative to normal cells that showed P-values less than 0.05.

We also interrogated the Oncomine database for the 5 most discriminating anti-apoptotic genes in the SYK induction model for their expression in leukemia and lymphoma patients. This revealed that for 20/31 comparisons at least one of the anti-apoptotic genes was represented in the gene signature. Enrichment of at least 3 out of the 5 genes was observed in 6 comparisons (B-lineage ALL, Burkitt’s lymphoma, centroblastic lymphoma, CLL, DL-BCL, hairy cell leukemia). Gene expression changes for centroblastic lymphoma, Burkitt’s lymphoma, DLBCL and hairy cell leukemia were enriched for both the anti-apoptotic and STAT3 target genes. One study demonstrated upregulation of SYK, STAT3, one STAT3 target gene and 3 anti-apoptotic genes for CLL diagnosis. Hierarchical two-way clustering (average distance metric for over expressed genes) of the Oncomine data exhibited a subcluster of diagnostic classes for Activated B-Cell-Like DLBCL and Germinal Center B-Cell-Like DLBCL that over expressed SYK, DAD1, HSPA5 and CYR61 and additional 2 classes (CLL and DLBCL) that overex-pressed both SYK and CYR61 (Figure 1). Six comparisons revealed a cluster of cases with increased expression of GCLC and SPRY2 (2 studies for CLL, B-Lineage ALL, Mantle Cell Lymphoma, Burkitt’s Lymphoma and DLBCL). One study that focused on childhood B-lineage ALL (Andersson_Leukemia) exhibited up regulation of DAD1 and TCF7L2 in addition to GCLC and SPRY2.

**Figure 2:**
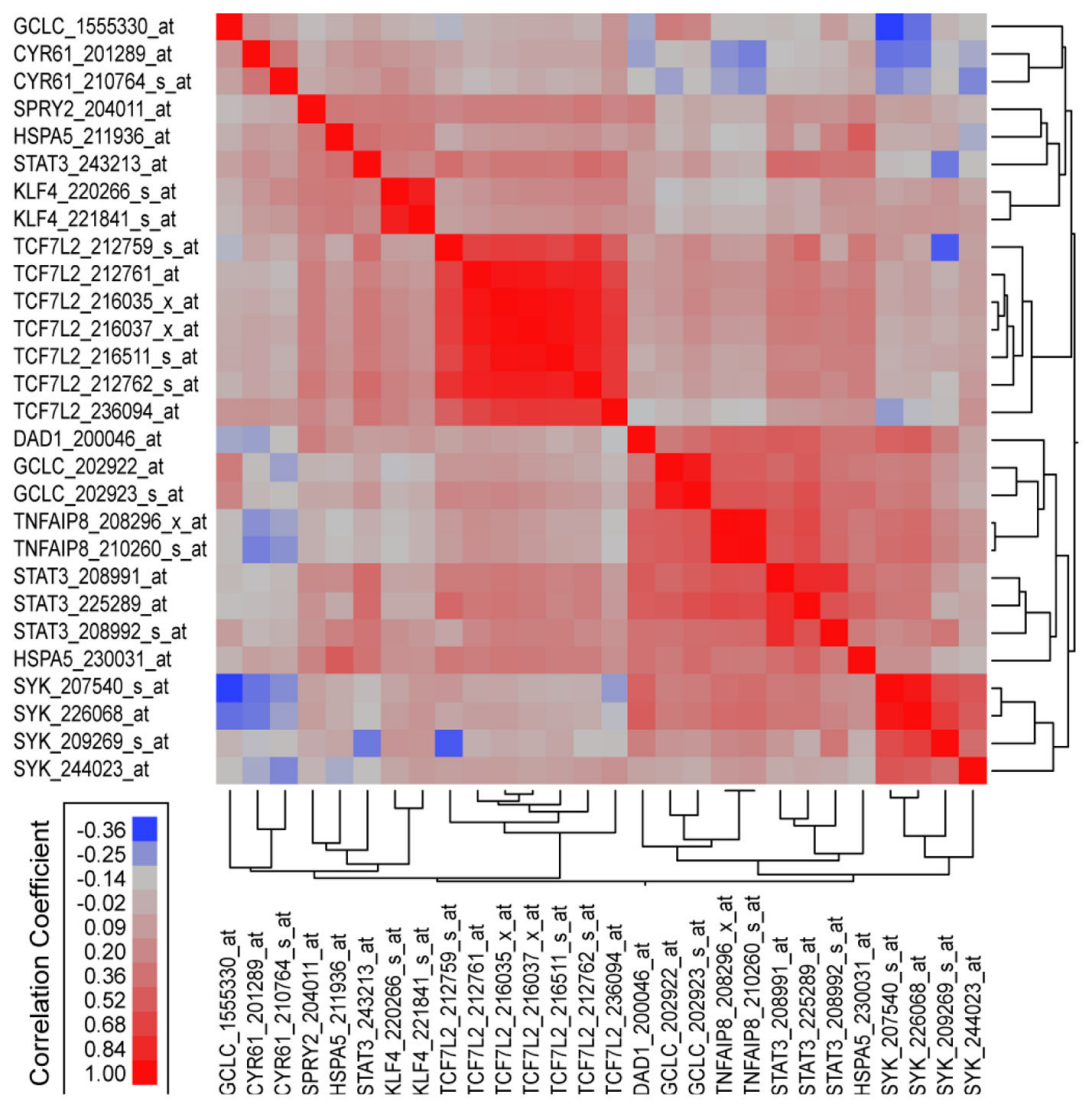
Correlations of the SYK Gene Signature in B/T-lineage Acute Lymphoblastic Leukemia Patients with SYK and STAT3 expression using the RMA Normalized Database obtained from 3 studies. Publically available gene expression datasets from 3 studies examining newly diagnosed B-lineage ALL (GSE1187 (N = 207), GSE13159 (N = 575), GSE13351 (N = 92)) and newly diagnosed T-lineage ALL (GSE13159 (N = 174), GSE13351 (N = 15)) were normalized using Robust Multi-array Averaging (RMA) method to compare expression profiles of SYK, STAT3 and SYK dependent STAT3 (KLF4, SPRY2, CYR61) and anti-apoptotic pathways (DAD1, GCLC, HSPA5, TCF7L2, TNFAIP-8). Pairwise correlations (Pearson coefficients) were performed for 28 probesets using the RMA normalized database. Correlation coefficients (r) were determined between all probeset pairs and two-way hierarchical agglomerative cluster analysis was applied to the matrix of correlation coefficients for both rows and columns of probeset identifications using the average distance metric to organize correlated expression profiles. Highly significant (P < 0.00001) correlations were observed for 234 out of the 378 possible pairs for probesets representing SYK, STAT3, and SYK/STAT3 target genes. Majority of these highly significant correlations were probesets that exhibited positive correlations versus negative correlations between these probesets (192 positive correlations, 42 negative correlations).

We further utilized an RMA normalized dataset combined from 3 studies (GSE1187, GSE13159, GSE13351 (N = 1063 samples)) examining the gene expression profiles of B-lineage and T-lineage ALL cells to determine the correlation structure of the 28 probesets for SYK, STAT3, SYK-STAT3 targets and SYK-antiapoptotic targets across these samples. Highly significant (P < 0.00001) pairwise correlations were observed for 234 out of the 378 possible pairs for 28 probesets representing SYK, STAT3, and SYK/STAT3 target genes. Majority of these highly significant correlations were probesets that exhibited positive correlations versus negative correlations between these probesets (192 positive correlations, 42 negative correlations) and highly significant positive correlations were significantly over represented in this leukemia dataset normalized from 3 studies (192/278 (69%) positive vs 42/100 (42%) negative correlations, Fisher’s exact test, 2-tailed, P < 0.0001). Pearson correlation coefficients were organized using a clustering algorithm to reveal highly positively co-regulated sets of probesets (Figure 2) whereby 4 probesets for SYK, 3 probesets for STAT3, 2 probesets for TNFAIP8, 2 probesets for GCLC and 1 probeset for DAD1 formed a metacluster of highly correlated expression values across 1063 leukemia samples. One of the probesets for STAT3 (208992_s_at) exhibited positive correlation with 25 out of the 27 other probesets including all the SYK probesets and SYK-dependent anti-apoptotic probesets. Two pro-besets that were negatively correlated with STAT3_ 208992_at (CYR61_210764_s_at and CYR61_201289_ at) were positively correlated with STAT3_243213_at suggesting that all probesets for SYK-STAT3 targets and SYK dependent anti-apoptotic targets are positively correlated with at least one of the STAT3 probesets.

**Figure 3:**
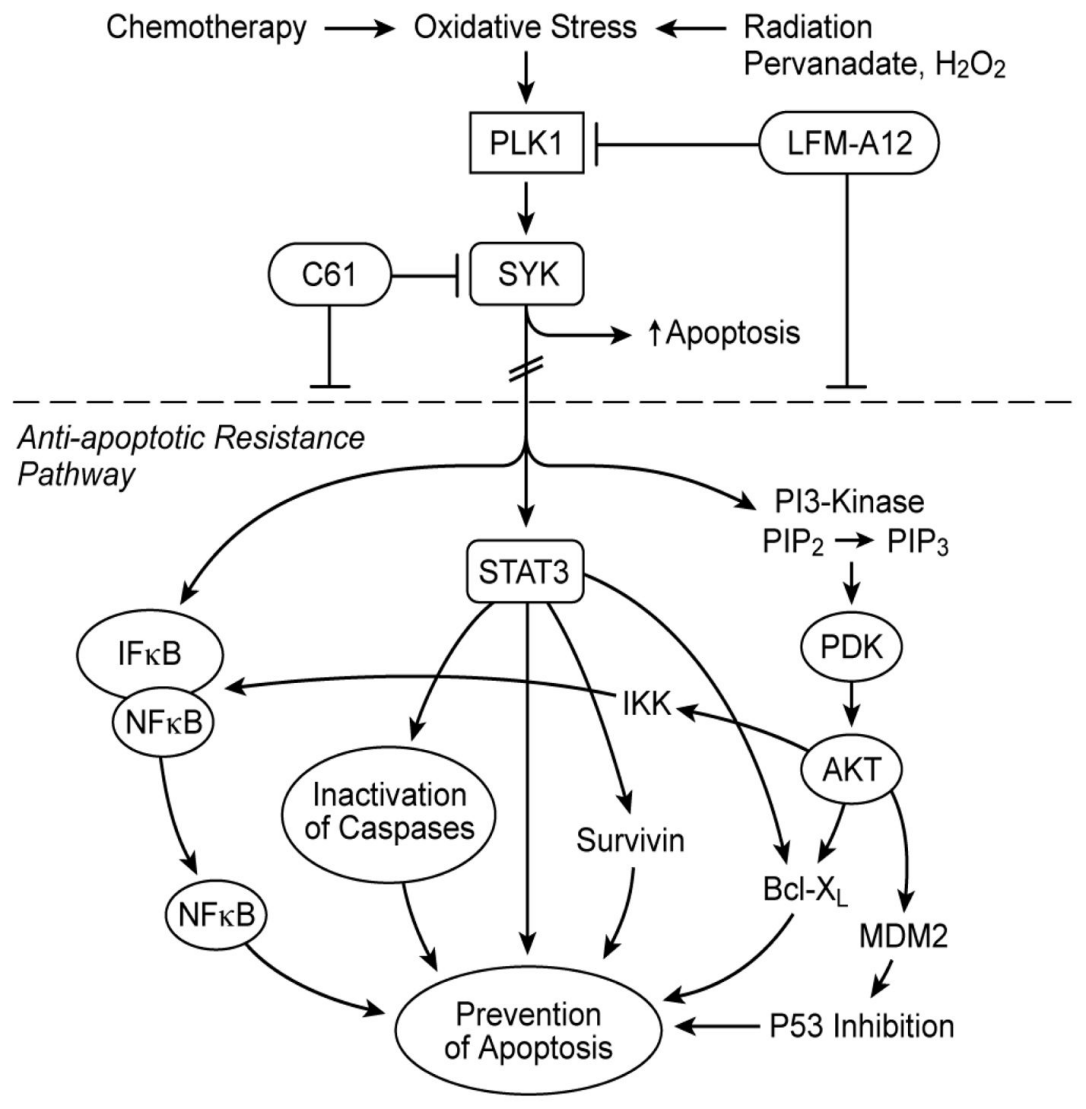
SYK as a Master Regulator of Apoptosis. Several chemotherapy drugs as well as radiation therapy exert their anti-cancer activity by inducing oxidative stress (OS). OS exposure of BPL cells triggers the activation of PLK1, which then activates SYK. Activation of SYK results in stimulation of the anti-apoptotic NFkB, STAT3, and PI3-K/AKT pathways and thus contributes to the frequently observed resistant phenotype of BPL cells. Inhibition of SYK with the small molecule inhibitor C61 directly inactivates SYK and causes apoptosis.

The identification of SYK as a master regulator of apoptosis controlling the activation of the PI3-K/AKT, NF*κ*B, and STAT3 pathways (Figure 3), three major anti-apoptotic signaling pathways in B-lineage leukemia/lymphoma cells, prompts the hypothesis that rationally designed inhibitors targeting SYK may overcome the resistance of malignant B-lineage lymphoid cells to apoptosis and thereby provide the foundation for more effective multi-modality treatment regimens for poor prognosis B-lineage leukemia and lymphoma patients. Therefore, SYK has emerged as a potential molecular target for treatment of B-lineage leukemias and lymphomas [9,12,13]. Constitutive activation and anti-apoptotic function of SYK have been documented for several B-lineage lymphoid malignancies, including BPL [9,13].

**Figure 4:**
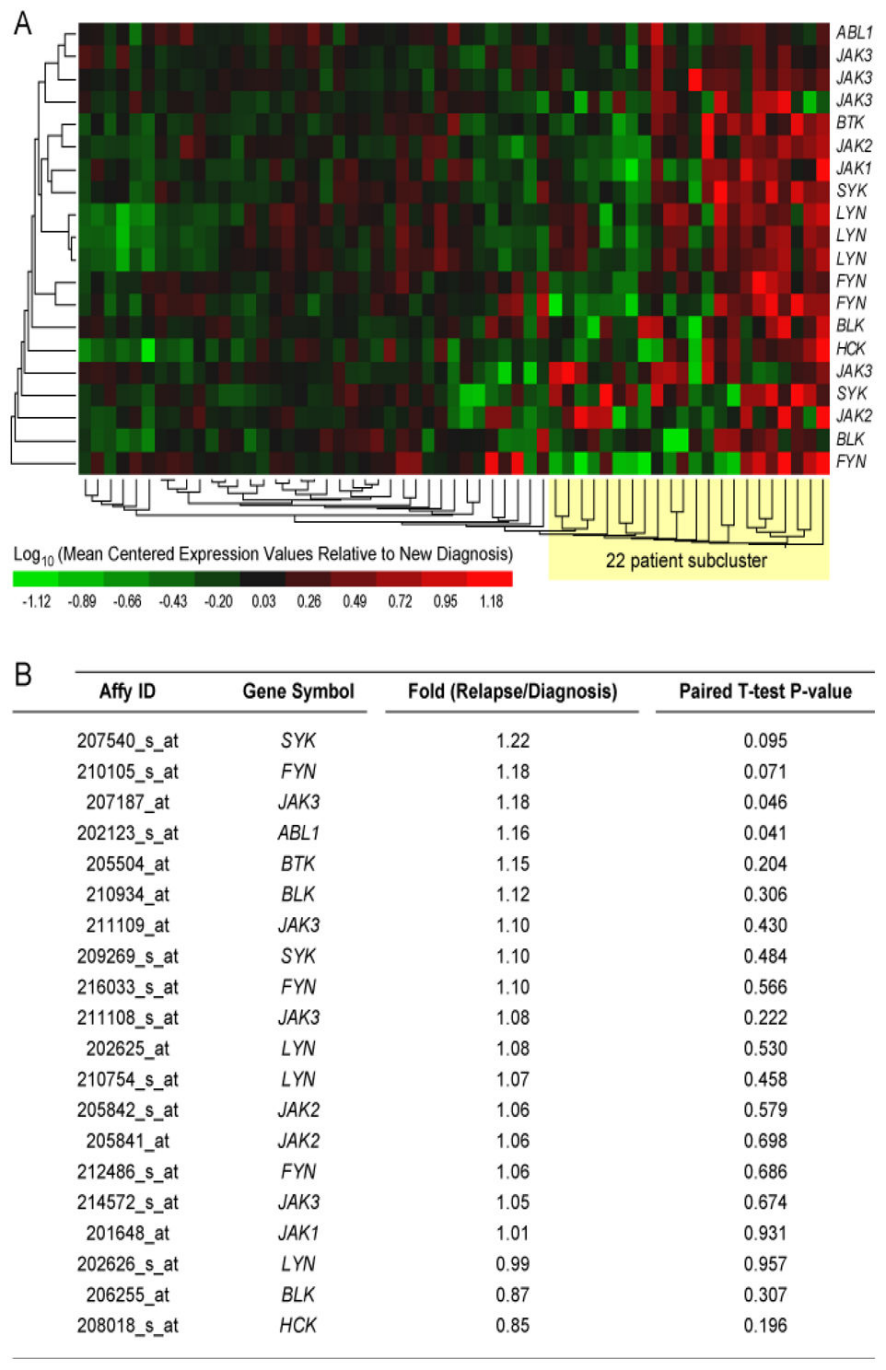
Kinase Gene Expression Profiles of Primary Leukemic Cells from Matched Pair Relapse vs. Diagnostic Bone Marrow Specimens of B-precursor ALL Patients. Gene expression values for leukemic cells in matched pair specimens taken from 59 BPL patients at diagnosis and then at relapse (combined from GSE3912, N = 32 and GSE 18497, N = 27). RMA-normalized values for the GSE18497 dataset and the MAS5-Signal intensity values for the GSE3912 dataset were log_10_ transformed and mean centered to the average value for the diagnosis samples for each gene transcript in each study. A two-way agglomerative hierarchical clustering technique was used to organize expression patterns using the average distance linkage method such that genes (rows) having similar expression across patients and patients with similar gene expression profiles were grouped together (average distance metric). The heat map represents the color-coded expression value for 59 matched pair diagnostic and relapse samples reported as the mean centered expression value relative to log_10_ transformed diagnostic samples.

Gene expression profiling of primary leukemic cells from matched pair relapse vs. diagnosis bone marrow specimens of 59 patients with BPL who relapsed showed similar expression levels for SYK (Figure 4) (Fold-Difference Relapse vs Diagnosis = 1.22/1.10, P = 0.095/0.484). Therefore, SYK is suited to serve as a molecular target for therapy against newly diagnosed as well as relapsed BPL. Expression profiles for *SYK* and *BTK* transcripts were highly correlated forming a subcluster in the hierarchical cluster representation. A subset of 22 patients exhibited signifycant increases in expression levels of *SYK* (2 transcripts; 1.84 fold, P = 0.038 (207540_s_at); 2.42 fold, P = 0.011 (209269_s_at)) and *BTK* (1.86 fold, P = 0.022 (205504_at)) at the time of relapse.

**Figure 5:**
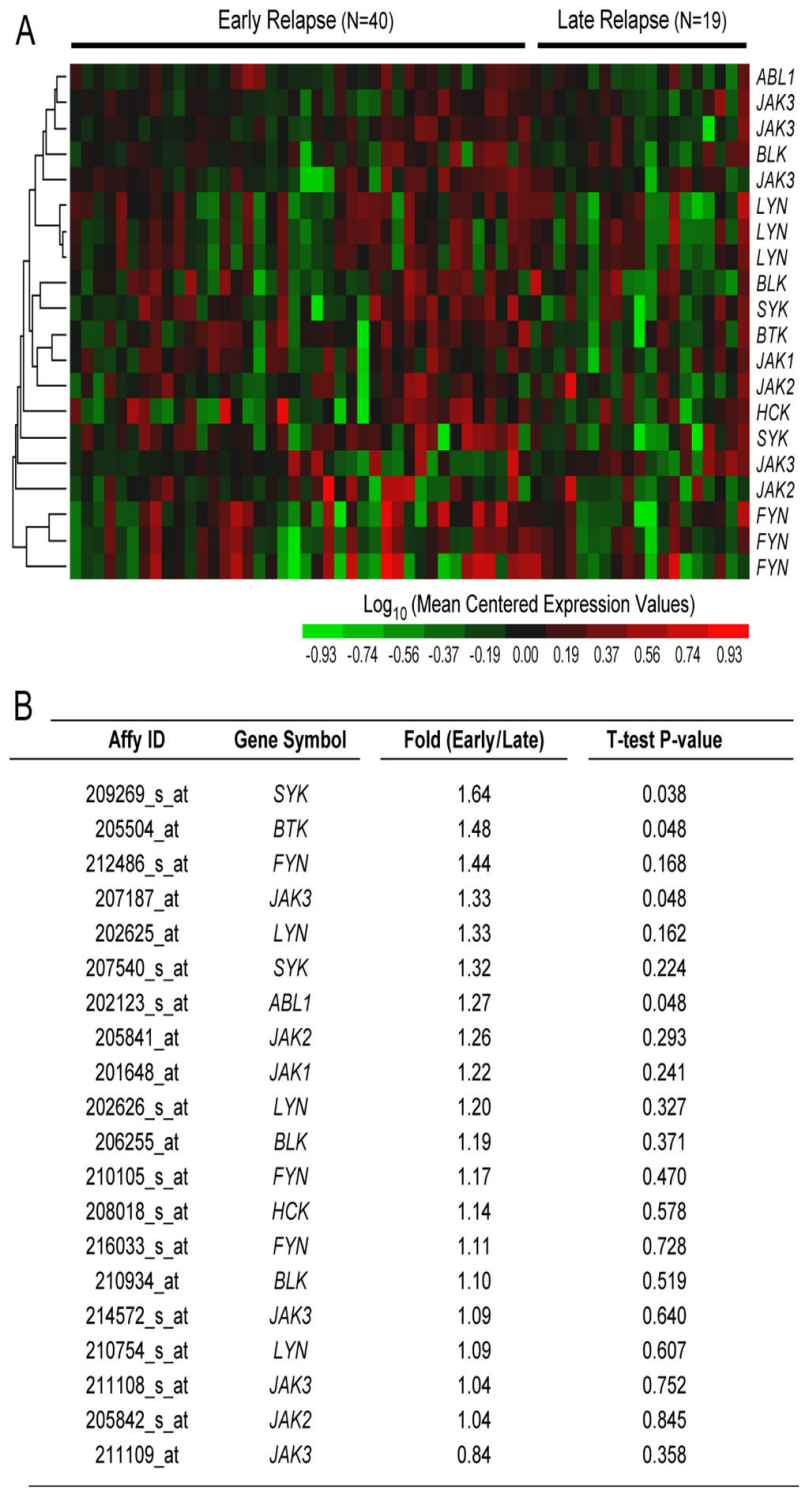
Kinase Gene Expression Profiles of Primary Leukemic Cells from Diagnostic Bone Marrow Specimens of B-precursor ALL Patients Who Experience an Early vs. Late Relapse. Gene expression values for primary leukemic cells in diagnostic specimens from BPL patients who experienced an early (N = 40; time to relapse < 36 months) versus late relapse (N = 19; time to relapse ≥ 36 months).

Intriguingly, comparison of *SYK* expression levels in primary leukemic cells in diagnostic specimens from patients who experienced an early (N = 40; time to relapse < 36 months) versus late relapse (N = 19; time to relapse ≥ 36 months) revealed higher expression levels for early relapse cases (Fold difference Early vs. Late Relapse: 1.64, P = 0.038, Figure 5), suggesting that *SYK* may be clinically useful both as a biomarker and molecular target for subpopulations of BPL patients who are at high risk for treatment failure and early relapse on standard chemotherapy regimens.

The identification of SYK as a regulator of the anti-apoptotic STAT-3 response to oxidative stress prompts the hypothesis that tyrosine kinase inhibitors targeting SYK may overcome the resistance to oxidative stress-induced apoptosis and thereby provide the foundation for more effective multi-modality radiochemotherapy and TBI regimens for poor prognosis BPL patients undergoing hematopoietic SCT. This hypothesis is strongly supported by the documented ability of a SYK kinase inhibitor to markedly enhance OS-induced apoptosis in primary leukemic cells from radiation-resistant ALL patients [11].

## 4. A New Nanomedicine Candidate Targeting the SYK-STAT3 Molecular Complex in Leukemic Stem Cells

The small molecule compound 1,4-bis(9-O-dihydroquinidyl) phthalazine/hydroquinidine1,4-phathalazinediyldiether (C61) is a substrate binding site inhibitor of SYK [11]. C61 is a structurally symmetrical molecule, which has a unique shape and a size not compatible for binding to the ATP binding site of the SYK kinase domain. The molecular volume of C61 (766 Å^3^) is larger than the available 686 Å^3^ volume of the binding pocket within the ATP binding site [11]. C61 has five individual molecular ring fragments representing functional analogs of five amino acid residues, resembling that of a tyrosine (Y)-containing pentapeptide (GDYEMN), which contains the DYE motif most favored by the protein substrate-binding region (P-Site) of SYK [11]. C61 has a molecular surface of 670 Å^2^, about half of which is buried by the surrounding protein residues. The occupation of the SYK P-Site by C61 results in competitive inhibition of substrate binding and thereby blocks the SYK signaling pathway. ATP binding site inhibitors of kinases are generally known to have off-target effects [10]. As a substrate-binding site inhibitor of SYK, C61 does not inhibit other kinases [11]. Inhibitors such as C61 are being designed as a new class of kinase inhibitors with greater selectivity [10]. In contrast to its potent nanomolar SYK inhibitory activity, even at high micromolar concentrations, C61 did not inhibit the enzymatic activity of EGF receptor kinase, TEC family tyrosine kinase BTK, SRC family tyrosine kinase HCK, SRC family tyrosine kinase LYN, Janus family tyrosine kinases JAK1, JAK2, JAK3, or the Insulin receptor kinase [11]. C61 is the first inhibitor targeting the P-site of SYK and the first apoptosis-promoting anti-cancer drug candidate in the cinchona alkaloid compound class. C61 inhibited SYK at nanomolar concentrations in both cell-free kinase assays using recombinant SYK as well as in cellular kinase assays using the BPL cell line NALM-6 [11,13]. Notably, treatment with C61 at dose levels 10-times lower than those found to be safe and nontoxic in cynomolgus monkeys, was capable of destroying > 99.9% of clonogenic B-lineage ALL cells *in vivo* and thereby improved the event-free survival outcome of SCID mice challenged with otherwise invariably fatal doses of human leukemic B-cell precursors in each of 3 different xenograft models of chemotherapy-resistant human B-lineage ALL [13]. C61 causes apoptosis in BPL cells that are resistant to standard chemotherapy [10].

Liposomal nanoparticle (LNP) formulations of active anti-cancer drugs may help design innovative treatments by contributing to reduced toxicity and improved pharmacodynamics of the drugs used as their payload [17–20]. LNPs have been coated with polyethylene glycol (PEG) (*i.e*., PEGylated) in an attempt to render them resistant to protein adsorption, improve their circulation half-life, reduce their renal clearance, and stabilize them against agglomeration during storage and in biological environments [18].

We therefore designed and produced a LPN formulation of C61. This nanomedicine formulation of C61 exhibited a uniquely favorable pharmacokinetics and safety profile in mice that was markedly superior to its salt formulation, induced apoptosis in radiation-resistant primary BPL blasts as well as BPL xenograft cells, killed the leukemic stem cell fraction of BPL blasts, and was effective in xenograft models of human BPL [17]. These proof-of-principle studies establish SYK as a new molecular target for personalized nanomedicine therapy of BPL. Further development of C61-LNP for a nanotechnology-enabled delivery of C61 to leukemia cells may provide the foundation for new and effective treatment strategies against therapy-refractory BPL.

## 5. Experimental Section

### Bioinformatics and Statistical Analysis of Gene Expression Profiles

Gene Pattern (http://www.broadinstitute.org/cancer/software/genepattern) was used to extract expression values for lymphoid kinase genes obtained from matched pair bone marrow specimens obtained from ALL patients at the time of initial diagnosis (1st specimen) and then at first relapse. Matched pair expression values were taken from 59 BPL patients at diagnosis and then at relapse combined from GSE3912 (N = 32) and GSE 18497 (N = 27). To determine the differential expression of each gene, Paired T-tests were performed for the combined mean centered values from GSE3912 and GSE18497 datasets (P < 0.05 deemed significant). Comparison of Early (N = 40; <36 months) versus Late (N = 19; ≥36 months) relapse subsets for newly diagnosed patients was performed to identify potential biomarkers for early relapse (2-sample T-test). Gene expression profiles were analyzed using standard bioinformatics tools, including one-way and two-way hierarchical clustering techniques. The heat map represents the color-coded expression value reported as mean centered expression level relative to log_10_ transformed diagnostic samples.

We compiled gene expression datasets from 3 studies examining newly diagnosed BPL/B-lineage ALL (GSE-1187 (N = 207), GSE13159 (N = 575), GSE13351 (N = 92)) and newly diagnosed T-lineage ALL (GSE13159 (N = 174), GSE13351 (N = 15)) to compare expression profiles of SYK, STAT3 and SYK dependent STAT3 (KLF4, SPRY2, CYR61) and anti-apoptotic pathways (DAD1, GCLC, HSPA5, TCF7L2, TNFAIP8) previously identified in our studies. To enable comparison of samples across studies, a normalization procedure was applied to the merged files from the 3 datasets (CEL files). Perfect Match (PM) signal values for probesets were extracted utilizing raw CEL files matched with probe identifiers obtained from the Affymetrix provided CDF file (HG-U133_Plus_2.cdf) implemented by Aroma Affymetrix statistical packages ran in R-studio environment (Version 0.97.551, R-studio Inc., running with R 3.01). The PM signals were quantified using Robust Multiarray Analysis in a 3 step process including RMA background correction, quantile normalization, and summarization by Median Polish of probes in a probeset across 1063 samples (RMA method adapted in Aroma Affymetrix). Normalization across all 3 studies and 1063 samples was achieved using a two-pass procedure. First the empirical target distribution was estimated by averaging the (ordered) signals over all arrays, followed by normalization of each array toward this target distribution. Pairwise correlations were performed for 28 probesets using the RMA normalized database. Correlation coefficients (r) were determined between all probeset pairs and hierarchical cluster analysis was applied to the matrix of correlation coefficients for both rows and columns of probeset identifications using the average distance metric to visualize subclusters of expression profiles (JMP Software, SAS, Cary, NC).

## 6. Conclusion

SYK is a master regulator of apoptosis controlling the activation of the PI3-K/AKT, NF*κ*B, and STAT3 pathways, three major anti-apoptotic signaling pathways in B-lineage leukemia/lymphoma cells. Gene expression profiling of primary leukemic cells from matched pair relapse vs. diagnosis bone marrow specimens of patients with BPL who relapsed showed similar expression levels for SYK. Therefore, SYK is suited to serve as a molecular target for therapy against newly diagnosed as well as relapsed BPL. Intriguingly, comparison of *SYK* expression levels in primary leukemic cells in diagnostic specimens from patients who experienced an early (N = 40; time to relapse < 36 months) versus late relapse (N = 19; time to relapse ≥36 months) revealed higher expression levels for early relapse cases, suggesting that *SYK* may be clinically useful both as a biomarker and molecular target for subpopulations of BPL patients who are at high risk for treatment failure and early relapse on standard chemotherapy regimens. Preclinical proof-of-concept studies have demonstrated that SYK can be useful as a molecular target for nanomedicine therapy in ALL. The liposomal nanoparticle formulation of the SYK P-site inhibitory small molecule compound C61 shows potential as an anti-leukemic nanomedicine candidate.
